# Grain Filling Characteristics and Their Relations with Endogenous Hormones in Large- and Small-Grain Mutants of Rice

**DOI:** 10.1371/journal.pone.0165321

**Published:** 2016-10-25

**Authors:** Weiyang Zhang, Zhuanqin Cao, Qun Zhou, Jing Chen, Gengwen Xu, Junfei Gu, Lijun Liu, Zhiqin Wang, Jianchang Yang, Hao Zhang

**Affiliations:** Jiangsu Key Laboratory of Crop Genetics and Physiology, Co-Innovation Center for Modern Production Technology of Grain Crops, Yangzhou University, Yangzhou, China; University of Manitoba, CANADA

## Abstract

This study determined if the variation in grain filling parameters between two different spikelet types of rice (*Oryza sativa* L.) is regulated by the hormonal levels in the grains. Two rice mutants, namely, a large-grain mutant (AZU-M) and a small-grain mutant (ZF802-M), and their respective wild types (AZU-WT and ZF802-WT) were grown in the field. The endosperm cell division rate, filling rate, and hormonal levels: zeatin + zeatin riboside (Z+ZR), indo-3-acetic acid (IAA), polyamines (PAs), and abscisic acid (ABA) were determined. The results showed that there was no significant difference between the filling and endosperm cell division rates. These rates were synchronous between the superior and inferior spikelets for both mutants. However, the abovementioned parameters were significantly different between the two spikelet types for the two wild types. The superior spikelets filled faster and their filling rate was higher compared to the inferior ones. Changes in the concentrations of plant hormones were consistent with the observed endosperm cell division rate and the filling rate for both types of spikelets of mutant and wild type plants. Regression analysis showed a significant positive correlation between cell division and filling rates with the concentrations of the investigated hormones. Exogenous chemical application verified the role of ABA, IAA, and PAs in grain filling. The results indicate that poor filling of inferior spikelets in rice occurs primarily due to the reduced hormone concentrations therein, leading to lower division rate of endosperm cells, fewer endosperm cells, slower filling rate, and smaller grain weight.

## Introduction

Grain filling is the final stage in rice growth at which the fertilized ovaries develop into caryopses [1]. The filling rate and extent determine the final weight of the rice grain and these factors are vital to grain yield and quality. There is considerable demand for further increase in the rice yield, and improving the filling of rice is now a prominent challenge [2, 3]. Generally, the upper or superior spikelets, flower earlier, fill faster, and produce larger and heavier grains. On the other hand, lower or inferior spikelets flower later, and are either sterile or fill slowly to produce grains. The upper and lower spikelets are located on apical primary and proximal secondary branches, respectively. Many theories have been proposed to explain why lower spikelets fill poorly, including source limitation [4], limitation in the sink size [5], hormonal imbalance [6, 7], a reduced activity or gene expression level of enzymes involved in sucrose-to-starch conversion [8, 9], and assimilate transportation limitation [10, 11]. However, the mechanism responsible for the observed variations of in the rate at which grains fill in these two types of spikelets remains to be fully elucidated.

Endogenous plant growth regulators have been reported to function in many physiological processes, such as cell division, morphogenesis, embryogenesis, responses to environmental stressors, fruit set, fruit growth, and senescence [6, 12–14]. Cytokinins are the key regulators of plant growth and development, and function in many processes such as division of cells, biogenesis of chloroplasts, differentiation of the bud and root, initiation and growth of shoot meristem, tolerance to stress, and senescence [15, 16]. As biologically important cytokinins in most of the higher plants are (Z)-type cytokinins: Zeatin (Z) and zeatin riboside (ZR). Z + ZR in developing rice and wheat (*Triticum aestivum*) grains were found to have large transient increases after pollination, coinciding with the period of seed setting and maximum cell division of the endosperm [17]. Furthermore, it was suggested that auxins and gibberellins (GAs) may contribute to the regulation of grain development. Eeuwens and Schwabe found that compounds similar to GA were present with the highest concentration in the liquid endosperm of pea during rapid pod elongation [18]. Suzuki et al. found relatively high GA_1_ and GA_19_levels in the large panicle of rice just prior to and at the time of anthesis [19]. In rice grains, poor filling was shown to have an association with low concentrations of indole-3-acetic acid (IAA) and ABA [20, 21]. Lu and Setter [22] studied maize (*Zea mays*) kernels and observed an abrupt rise in the IAA concentrations in the endosperm approximately 10 days after pollination, coinciding with increased DNA content in each nucleus. Previous study has suggested that the ABA content is greater in large grains than in smaller ones during filling; furthermore, this parameter has been found to be positively correlated with the filling rate at the early stages of filling in wheat [23]. The polyamines (PAs) spermidine (Spd) and spermine (Spm) and their diamine obligate precursor putrescine (Put) have all been described as important regulators of plant growth. PAs were detected at significantly lower levels in aborting maize kernels than in normal maize kernels, and these low concentrations were associated with a low number of endosperm nuclei and low DNA content detected at the same timeframe [24]. Many studies have investigated the mechanism of filling and the differences between the two types of spikelets in rice varieties with different genetic backgrounds, such as rice varieties with large and small panicles; however, little is known about the cause of poor filling in inferior spikelets in rice varieties with similar genetic backgrounds.

The current investigation aimed to study the processes of division of endosperm cells; filling of rice grains; and changes in the levels of various hormones, namely, GAs, Z, ZR, IAA, ABA, and PAs, in both types of spikelets of two rice mutants and their respective wild types during the filling stage and to then identify whether the hormones regulate these variations in filling between the different types of spikelets. We observed the effects of chemical regulators on the hormonal levels in the grains and on their filling characteristics.

## Methods

### Materials and culture conditions

The experiment location was a farm in Yangzhou University, China (32° 30′N, 119° 25′E) from May to October 2012 and during the same period in 2013. The rice was grown in a sandy loam soil (Typic fluvaquents, Etisols [US taxonomy]) with organic matter (24.4 g·kg^–1^), alkali hydrolysable N (106 mg·kg^–1^), Olsen-P (34.5 mg·kg^–1^), and exchangeable K (68.5 mg·kg^–1^). The total precipitation during the filling period was 60 mm and 65 mm in 2012 and 2013 respectively. The mean solar radiation during filling measured at a weather station close to the experimental site was18.0 MJ m^–2^·d^–1^in 2012 and 17.7 MJ m^–2^·d^–1^ in 2013.

One large-grain mutant (AZU-M) and one small-grain mutant (ZF802-M) and their respective wild types (AZU-WT and ZF802-WT), all of which were field grown, were used in this study. AZU-WT is an inbred japonica cultivar, and ZF802-WT is an inbred indica cultivars. The large-grain mutant (AZU-M) and small-grain mutant (ZF802-M) were generated by radiation from AZU and ZF802, respectively. The 1000-grain weight of AZU-M, AZU-WT, ZF802-M and ZF802-WT are 62.94±0.61 g, 29.43±0.32 g, 11.98±0.18 g, and 23.75±0.29 g, respectively. All the cultivars and mutants were bred by Zhejiang Academy of Agricultural Sciences, China. At the sixth generation generated by radiation, both mutants showed phenotypic stability and were used for studying grain filling characteristics [25]. The seeds of both mutants used in the present experiment were the 7th and 8th generation. All the cultivars have similar growth periods ranging from 138 to 141 days from sowing to physiological maturity. Seedlings were sowed in the field on 14 May and transplanted on 15 June at a hill spacing of 0.2 m×0.2 m with one seedling per hill. The plot dimension was 5.6 m×3.5 m. Each of the genotypes had three plots as repetitions. Nitrogen (120 kg·ha^–1^ as urea), phosphorus (30 kg·ha^–1^ as single superphosphate) and potassium (40 kg·ha^–1^ as KCl) were applied before transplanting. Urea served as the nitrogen source and was applied at the mid-tillering (36 kg·ha^–1^) and panicle initiation (60 kg·ha^–1^) stages and at 18–20 days before heading (24 kg·ha^–1^). The water, weeds, insects, and diseases were controlled as required to avoid yield loss. All the cultivars (50% of the plants) headed on 18–20 August, and were harvested on 1–2 October, 2012 and 2013. Except for drainage at the end of tillering (11–15 July), the field was continuously maintained under 1–2 cm of water during the whole growth period.

### Sampling

A total of 400 panicles that headed on the same day were chosen and tagged for each plot. Then, we noted the date of flowering and the spikelet position of each spikelet on some of the tagged panicles. Approximately 10–12 tagged panicles from each plot were sampled at three-day intervals from anthesis to maturity. Half of the sampled grains were frozen in liquid nitrogen for 2 min, and then stored at –80°C for hormone measurements. The remaining half were dried at 70°C to a constant weight for 72 h, dehulled, and then their weights were weighed. We sampled five tagged panicles per plot at three-day intervals from the period of anthesis to 42 DPA in order to determine the endosperm cell number. We then fixed 10–12 rice grains with a small hole cut on the edge of a hull in Carnoy’s solution (absolute ethanol: glacial acetic acid: chloroform = 9:3:1, v/v/v) for 48 h, after which the grains were stored in 70% (v/v) ethanol until they could be evaluated for endosperm cell number. The superior spikelets located on apical primary branches that flowered on the first 2 days and the inferior ones located on proximal secondary branches that flowered on the last 2 days within a panicle were separated from the sampled panicles. There was a difference of about 3–5 days in the flowering date between the two types of spikelets within a panicle for all the four rice variants in this study.

### Isolation and counting of endosperm cells

Endosperm cells were isolated and counted according to the method used by Singh and Jenner [25], with some modifications. In brief, fixed grains were first dehulled, transferred into 50% and 25% (v/v) ethanol. The grains were transferred to distilled water and allowed to sit for 5–7 h before endosperm dissection. Endosperm isolation was carried out under a dissecting microscope, after which it was dyed by incubation with Delafield’s haematoxylin for 24–30 h, washed many times with distilled water, hydrolyzed in 0.1% (w/v) cellulase (No. c-2415; Sigma, St Louis, MO, USA) solution (pH 5.0) at 40°C for 4–6 h, then oscillated. The isolated endosperm cells were diluted to 2–10 mL depending on their developmental stage. From these samples, 8–10 samples (20 μL each) were transferred to a counting chamber (area, 1 cm^2^). The endosperm cell number in ten fields per counting chamber was then observed using light microscopy and recorded. The number of nuclei observed at 2–4 DPA was counted as the endosperm cell number. We then calculated the total number of cells in each endosperm according to the method of Liang et al. [26]. We examined a total of eight endosperms per genotype for each measurement. The division of the endosperm cells, and the filling process were fitted to the Richards’s growth equation (Richards 1959) according to the method described by Zhu et al. [27]:

$$W=\frac{A}{{\left(1+Be^{-kt}\right)}^{\frac{1}{N}}}$$ (1)

The rate of division or the rate of filling (*R*) of endosperm cells was calculated as the derivative of Eq 1

$$R=\frac{AkBe^{-kt}}{N{\left(1+Be^{-kt}\right)}^{\frac{\left(N+1\right)}{N}}}$$ (2)

where *W* denotes cell number or grain weight, *A* denotes the maximum cell number/final grain weight; *t* denotes the time after anthesis (days); and *B*, *k*, and *N* are the regression coefficients. The period of active cell division/filling is defined as the time interval taken for *W* to change from 5% (*t*_1_) to 95% (*t*_2_) of *A*. The average rate of cell division or filling during this period was calculated from *t*_1_ to *t*_2_.

### Extraction, purification, and quantification of hormones

The various hormones studied here were extracted and purified using the methods of Bollmark et al. [28] and He [29] with modifications. Briefly, 50–80 frozen dehulled grains were ground in an ice-cold mortar containing 5–10 mL of 80% (v/v) methanol extraction medium with 1 mM butylated hydroxytoluene as an antioxidant. The extract was incubated at 4°C for 4 h and centrifuged at 1467 ×g for 15 min at 4°C. The supernatants were run in a Chromosep C_18_ column (C_18_ Sep-Park Cartridge, MA, USA), prewashed with 10 mL of 100% (v/v) and 5 mL of 80% (v/v) methanol. The obtained hormone fractions were dried under liquid nitrogen and used for enzyme-linked immunosorbent analysis (ELISA). Prior to ELISA, the fractions were prepared by dissolving in phosphate-buffered saline (PBS, 2 mL) containing 0.1% (v/v) Tween-20 and 0.1% (w/v) gelatin (pH 7.5).

The antigens, antibodies against the hormones, and IgG horseradish peroxidase (HRP) were all obtained from the Phytohormone Research Institute (China Agricultural University; [29]). The hormonal compounds analyzed in this study, namely, Z, ZR, IAA, GAs, and ABA, were quantified using ELISA according to the procedure described previously [30].

### Extraction and quantification of PAs

We estimated free PA fractions according to the method of Flores and Galston [31], and we analyzed soluble- and insoluble-conjugated fractions using the method of Liu et al. [32] with modifications. Briefly, we obtained samples containing 80–100 grains. The grains were mixed with 3–5 mL of 5% (v/v) perchloric acid (PCA) and then homogenized in a precooled mortar and pestle. The resultant homogenate was first incubated at 5°C for 1 h, followed by centrifugation at 25,000 ×g for 20 min. The resultant supernatant and residue were collected separately. Soluble-conjugated PAs were extracted by mixing 2-mL aliquots of the supernatant with 2 mL of 12 N HCl, and heating the mixture at 110°C for 18 h in flame-sealed glass ampoules. After acid hydrolysis, the HCl was evaporated by heating the tubes at 80°C, and the residues were resuspended in 0.5 mL of 5% (v/v) PCA. For insoluble-conjugated PAs, the residue was first rinsed four times with 5% PCA to remove any trace of soluble PA. Then, the pellet was dissolved in 2 mL of 1 N NaOH by vigorous vortexing. This mixture was centrifuged at 25,000 g for 20 min, and the supernatant was hydrolyzed as mentioned above.

In the non-hydrolyzed supernatant, hydrolyzed supernatant, and hydrolyzed pellet, the PAs were extracted using benzoyl chloride and quantified in a high-performance liquid chromatograph (Waters 2695 Separations Module; Waters, MA). Samples that had been redissolved in exactly 10 μL of methanol (60% v/v) were injected into a fixed 20-μL loop for loading onto a reverse-phase (C_18_) column ((4.6 by 250 mm^2^, particle size: 5 μm; Waters). The samples were eluted from the column with a flow rate of 0.6 mL·min^–1^at 25°C using a Perkin-Elmer series 410 pump Polyamine peaks were detected at an absorbance of 254 nm in a spectrophotometer (Perkin-Elmer LC-95). The soluble-conjugated PA concentration was calculated by subtracting the concentration of free PA in the non-hydrolyzed supernatant from the PAs in the hydrolyzed supernatant with 1,6-hexanediamine as the internal standard. The concentration of PA was determined as the average of three replicates per independent sample, and the value was expressed as nmol·g^–1^of fresh weight (FW).

### Final harvest

On both 2012 and 2013, all the plant samples were harvested by hand on 1–2 October. The grain yield was determined by measuring this parameter in all the plants within a 5-m^2^ are (excluding border plants) per plot, and the yield was then adjusted to a moisture content of 0.14 g of H_2_O/g FW. A total of 50 plants (excluding border plants) were sampled randomly from each plot, and the yield components, including the number of panicles·hm^–2^, proportion of filled grains, and grain weight were determined. Spikelet fertility was determined by averaging the results from 40 comparable mature panicles per plot and expressed as the percentage of aborted or sterile spikelets), partially filled, and fully filled grains compared to the total number of potentially fertilizable spikelets per panicle. The proportion of filled grains was measured as the number of filled grains (specific gravity≥1.06 g/ml) to the total number of spikelets. The following equation was used to calculate the number of spikelets in each panicle: 1000×grain yield·hm^–2^·(number of panicles in each hectare × the 1000-grain weight of filled grains). The total number of spikelets was determined by multiplying the number of panicles·hm^–2^ × the number of spikelets in each panicle.

### Chemical treatment

The two wild-type strains, AZU-WT and ZF802-WT, were used for the chemical application. Plants were cultivated in paddy fields under the abovementioned culture conditions. At the stage of early endosperm development (2–6 DPA), either 2 mM Put, 1 mM Spd, 1 mM Spm, or 5 mM of the SAM decarboxylase inhibitor methylglyoxal-bis (MGBG, guanylhydrazone) was applied to the panicles and either 20×10^−5^ M of IAA or 25×10^−6^ M of ABA were applied to the panicles of the two cultivars. The chemicals were applied daily for 5 days at the rate of 4 mL per panicle for each application. A writing brush dipped in the solution was used for the application, and 0.5% (v/v) Teepol (Fluka, Riedel-de-Haen, Germany) was used as the surfactant. All the solutions contained 0.1% ethanol (v/v) and 0.01% Tween-20 (v/v). The control plants were sprayed with the same volume of deionized water containing the same concentrations of ethanol and Tween-20. Each treatment was performed on 200 panicles each, with three replications per set. For all the chemical treatments, the spikelets were divided into superior and the inferior. Hormone concentrations in the inferior spikelets were measured at 12, 15, and 18 DPA. The endosperm cell number was measured at 4-day intervals from anthesis to 28 DPA, and grain weight was measured at 4-d intervals from anthesis to maturity.

### Statistical analysis

Statistical analyses of the results for variance were carried out using the SAS/STAT statistical analysis package (version 6.12, SAS Institute; Cary, NC, USA). Data from each sampling date were analyzed separately, and the resultant means were tested by the least significant difference at the *P*_0.05_ level (*LSD*_0.05_). Linear regression analysis was used to evaluate the relationships between the hormone concentrations in the grains and the endosperm cell division rate or filling rate. Since data from both the years showed similar tendencies, they were averaged.

## Results

### Grain yield, endosperm cell division, and filling

Table 1 presents the grain yield and its components in all the rice cultivars. The spikelet number per panicle showed no significant difference between the mutants and their wild-type variants. The 1000-grain weight of AZU-M was 62.94 g, which was 2.14 × the 1000-grain weight of AZU-WT (control); however, there were only 63% panicles, and < 50% of the grains were filled, and the grain yield was only 43% when compared with that of control plant (Table 1). Although there were significantly more panicles in ZF802-M than in the control, the1000-grain weight and percentage of filled grains in this mutant were only 50% and 54% of the control; consequently, the grain yield of this mutant was less than 33% that of the control (Table 1). The reduced fertilization rate (larger number of sterile spikelets) is an important reason for the reduced number of filled grains found in AZU-M and ZF802-M (Table 1).

**Table 1:** No. of panicles, spikelets per panicle, total spikelets, filled grains, 1000-grain weigh, sterile spikelets, and grain yield.

Cultivar	No. of panicles (×10^4^ hm^-2^)	Spikelets per panicle	Total spikelets (×10^6^ hm^-2^)	Filled grains (%)	1000-grain weight (g)	Sterile spikeles (%)	Grain yield (t hm^-2^)
AZU-WT	155.70 ± 5.11 a	211.34 ± 6.78 a	329.24 ± 20.27a	79.59 ± 2.62a	29.43 ± 0.32b	5.45 ± 0.17b	8.14 ± 0.73a
AZU-M	97.89 ±4.18b	218.49 ± 8.91 a	213.66 ±16.25b	26.2 ± 0.56b	62.94 ± 0.61a	65.7 ± 0.35a	3.52 ± 0.23b
ZF802-WT	223.39 ± 7.10b	136.84 ± 5.76 a	305.91 ± 20.07b	94.77 ± 1.74a	23.75 ± 0.29a	2.15 ± 0.03b	6.87± 0.56a
ZF802-M	296.18 ± 1.415a	124.13 ± 5.349 a	367.96 ± 15.46 a	51.5 ± 0.32b	11.98 ± 0.18b	40.5 ± 0.40a	2.26 ± 0.08b

Data are mean±SE of six independent measurements and different letters indicate statistical significance at the *P* = 0.05 level within the same genetic background cultivars.

**Fig 1:**
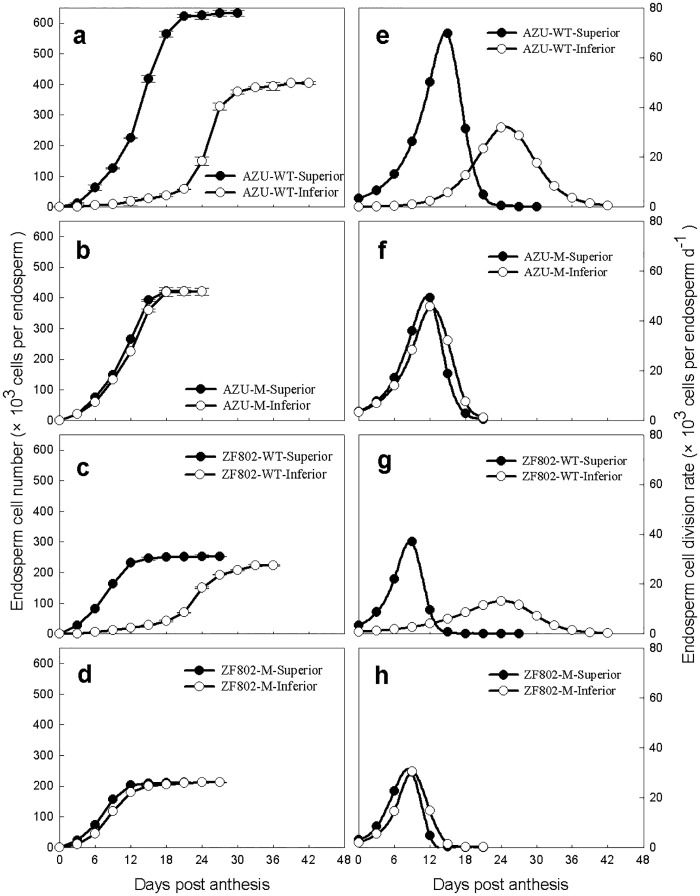
Endosperm cell number (a-d) and division rate (e-h) in both superior (closed circle) and inferior (open circle) spikelets of rice. The wild types, AZU-WT (a, e) and ZF802-WT (c, g), and their mutants, AZU-M (b, f) and ZF802-M (d, h) were field grown. *Vertical bars* represent ± SE of the mean (n = 6) where these exceed the size of the symbol.

Fig 1 presents a comparison of the progress of division and the rate of endosperm cells between both spikelet types. The maximum endosperm cell number and division rate was higher in the superior than in the inferior spikelets for both the wild-type varieties. The abovementioned parameters did not significantly differ between the two spikelet types for both the mutants. The maximum endosperm cell number in the spikelet types was higher in the wild-type varieties than in the mutants. The maximum endosperm cell division rate in the superior spikelets was higher in the wild-type varieties than in the mutant types. However, the maximum endosperm cell division rate was lower in the wild type than in the mutant types in the inferior spikelets (Fig 1a–1h).

**Fig 2:**
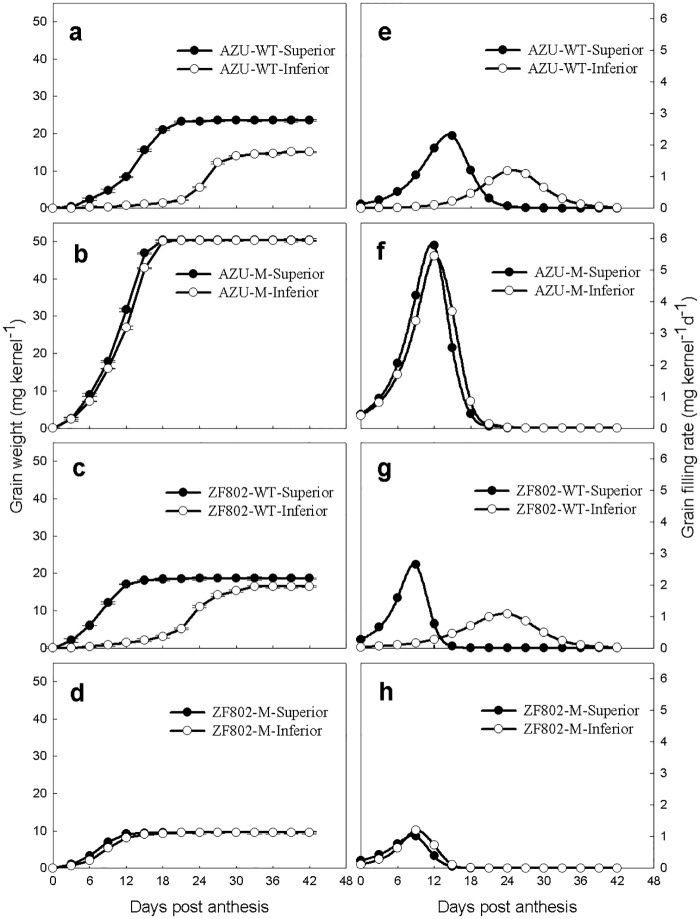
Grain weight (a-d) and grain filling rate (e-h) in both superior (closed circle) and inferior (open circle) spikelets of rice. The wild types, AZU-WT (a, e) and ZF802-WT (c, g), and their mutants, AZU-M (b, f) and ZF802-M (d, h) were field grown. *Vertical bars* represent ± SE of the mean (n = 6) where these exceed the size of the symbol.

Similar to the trend observed for endosperm cell number, the grain weight and maximum filling rate were higher in the superior than in the inferior ones for both wild types but not for the two mutant types. The weight and maximum filling rate for both types of spikelets were higher in AZU-M than AZU-WT, whereas an opposite trend was observed between ZF802-M and its wild type (Fig 2a–2h).

### Hormonal changes

**Fig 3:**
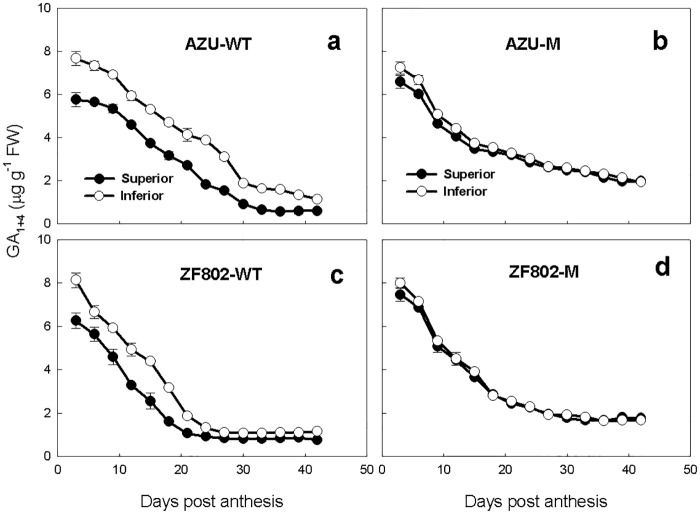
Changes in the concentrations of GAs (GA_1_+GA_4_) (a-d) in both superior (closed circle) and inferior (open circle) spikelets of rice. The wild types, AZU-WT (a) and ZF802-WT (c), and their mutants, AZU-M (b) and ZF802-M (d) were field grown. *Vertical bars* represent ± SE of the mean (n = 6) where these exceed the size of the symbol.

During grain filling, the concentration of GAs was highest at the early stage, declined with the process of filling (Fig 3a–3d). The concentration of GAs was significantly higher in the inferior spikelets than in the superior ones for the wild types, while such a difference was not significant for mutants or between a mutant and its wild type (Fig 3a–3d).

**Fig 4:**
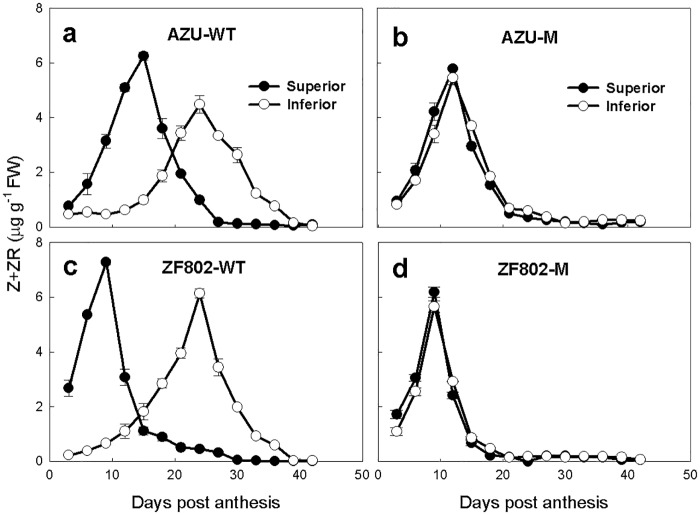
Changes in the concentrations of Z+ZR (a-d) in both superior (closed circle) and inferior (open circle) spikelets of rice. The wild types, AZU-WT (a) and ZF802-WT (c), and their mutants, AZU-M (b) and ZF802-M (d) were field grown. *Vertical bars* represent ± SE of the mean (n = 6) where these exceed the size of the symbol.

**Fig 5:**
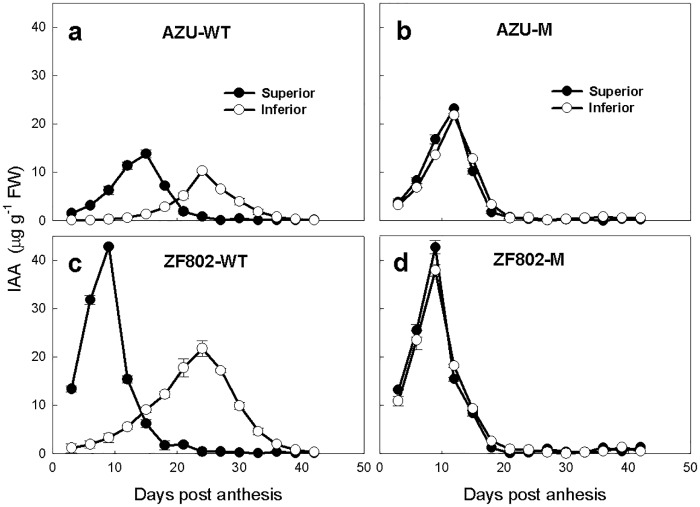
Changes in the concentrations of IAA (a-d) in both superior (closed circle) and inferior (open circle) spikelets of rice. The wild types, AZU-WT (a) and ZF802-WT (c), and their mutants, AZU-M (b) and ZF802-M (d) were field grown. *Vertical bars* represent ± SE of the mean (n = 6) where these exceed the size of the symbol.

**Fig 6:**
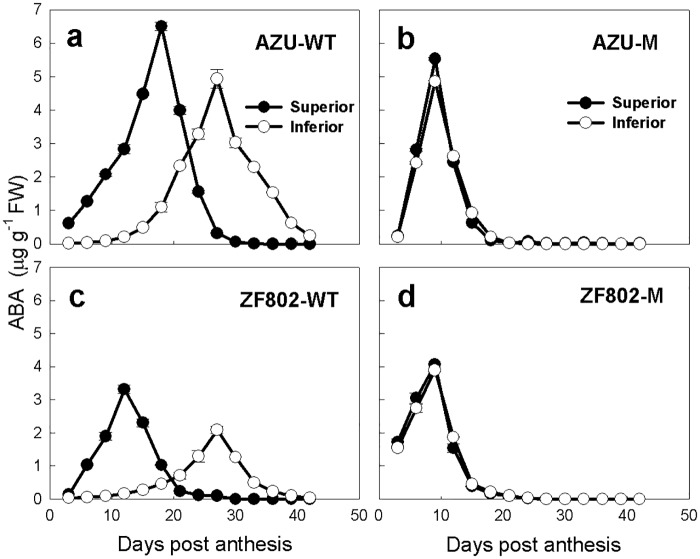
Changes in the concentrations of ABA (a-d) in both superior (closed circle) and inferior (open circle) spikelets of rice. The wild types, AZU-WT (a) and ZF802-WT (c), and their mutants, AZU-M (b) and ZF802-M (d) were field grown. *Vertical bars* represent ± SE of the mean (n = 6) where these exceed the size of the symbol.

Similarly, the Z+ZR, IAA, and ABA concentrations initially increased in the grains with progress in grain filling, peaked at 10–15 in the superior and 28–30 DPA in the inferior spikelets of the wild type, and at 9–12 DPA for both types of spikelets for mutant types, and these values decreased sharply thereafter (Figs 4–6). The peak values and concentrations of Z+ZR, IAA, and ABA were higher in the superior spikelets at the early filling stage (3–15 DPA); thereafter, these concentrations were lower than the corresponding values in the inferior spikelets for two wild types. For the mutant types, these values did not significantly differ between the two types of spikelets (Figs 4–6). Furthermore, the peak concentrations of Z+ZR in superior spikelets were higher in the wild types than in the mutants, and the peak concentrations of Z+ZR in inferior spikelets appeared earlier in the mutants than in their wild types (Fig 4). The peak concentrations of IAA were higher in inferior spikelets, and the peaks occurred earlier in mutants than in their wild types (Fig 5). The ABA peak values of the spikelets appeared earlier in mutants than in their wild types (Fig 6).

### Changes in polyamine concentrations

**Fig 7:**
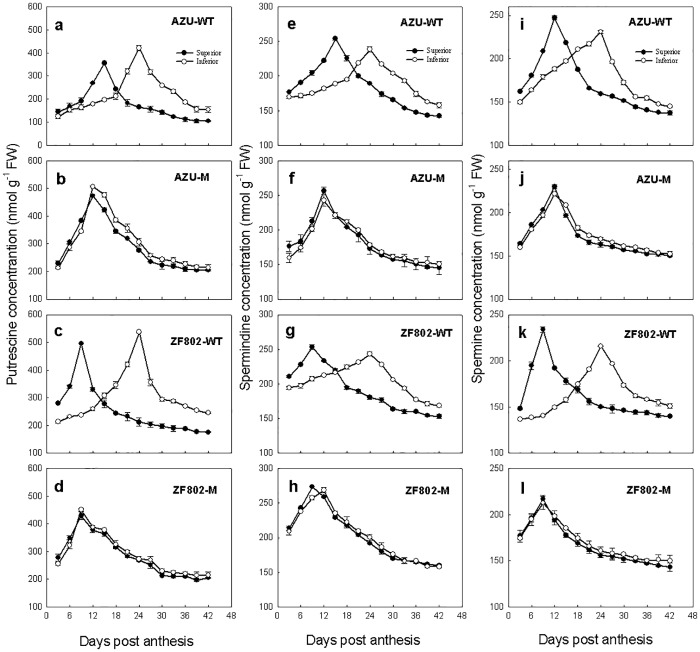
Changes in the concentrations of free putrescine (a-d), free spermidine (e-h) and free spermine (i-l) in both superior (closed circle) and inferior (open circle) spikelets of rice. The wild types, AZU-WT (a, e, i) and ZF802-WT (c, g, k), and their mutants, AZU-M (b, f, j) and ZF802-M (d, h, l) were field grown. *Vertical bars* represent ± SE of the mean (n = 6) where these exceed the size of the symbol.

The concentration of the free PAs (including Put, Spd, and Spm) increased in the grains during grain filling (Fig 7). PAs concentrations peaked at 9–15 for the superior spikelets and 9–24 DPA for the inferior spikelets, and sharp decreased thereafter (Fig 7). A similar trend was observed in the wild types, with superior spikelets exhibiting higher peak concentrations of free Spd and Spm and higher concentrations of free PAs at 3–12 DPA and relatively lower concentrations thereafter in comparison with the inferior spikelets. However, the free PA concentrations only slightly differed between the two types of spikelets, and they peaked at 12 DPA for both mutants. The wild-type varietties with higher endosperm cell division and filling rates were found to have higher maximum values of free Spd and free Spm (Fig 7e–7l). Furthermore, in both wild-types, the peak values of free Put was higher in the inferior spikelets than in the superior ones (Fig 7a–7d).

The levels of soluble- and insoluble-conjugated PAs did not change during the filling stage. The concentrations of these PAs were lower than those of free PAs, and the concentration of soluble-conjugated Put, Spd, and Spm ranged from 120–134, 59–71, and 43–52 nmol·g^–1^ FW, respectively, while the corresponding ranges of insoluble-conjugated PAs were 98–106, 65–81, and 26–35 nmol·g^–1^ FW, respectively. Moreover, the concentrations of soluble- and insoluble-conjugated PAs did not significantly differ between the two spikelet types or between the the wide types/mutants (data not shown).

### Correlation between filling rate and division rate with the hormone concentrations

**Fig 8:**
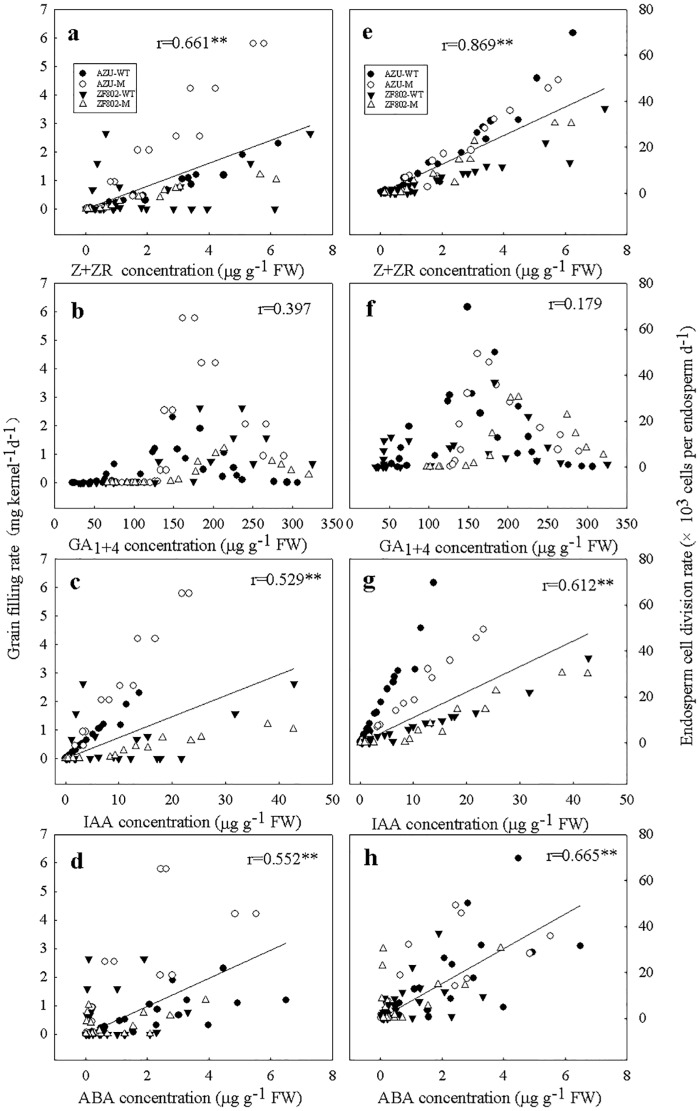
Correlations of grain filling rate (a-d) and endosperm cell division rate (e-h) with Z+ZR (a, e), GA_1+4_ (b, f), IAA (c, g) and ABA (d, h) concentrations in the rice spikelets. The data used for the calculation are from Figs 1–6. The asterisk (**) represents the statistical significance at the *P* = 0.01 level. (n = 112 for grain filling rate and n = 75 for endosperm celldivision rate).

**Fig 9:**
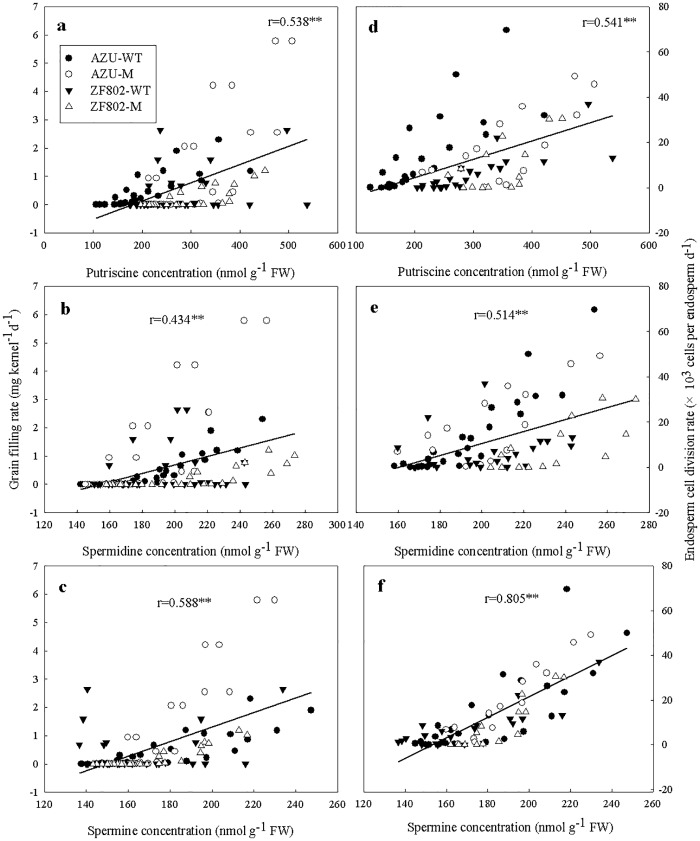
Correlations of grain filling rate (a-c) and endosperm cell division rate (d-f) with free-putrescine (a, d), permidine (b, e), and permine (e, f) concentrations in the rice spikelets. The data used for the calculation are from Figs 1, 2 and 7. The asterisk (**) represents the statistical significance at the *P* = 0.01 level. (n = 112 for grain filling rate and n = 75 for endosperm celldivision rate).

As shown in Figs 8 and 9, the filling rate and the endosperm cell division rate showed significant positive correlation with the Z+ZR, IAA, and ABA concentrations (Fig 8) and with the free Put, Spd, and Spm levels (Fig 9). However, there were no significant correlation between the filling rate and cell division rate and GA_1+4_ concentrations (Fig 8).

### Results of the chemical treatment

Chemicals that inhibit PAs and hormone synthesis and IAA and ABA were applied to panicles at 2–6 DPA in order to verify the role of hormones in the regulation of filling. As shown in S1 and S2 Tables, IAA treatment markedly increased the IAA, GA_1+4_, and Z+ZR concentrations, but decreased the ABA concentrations in the inferior spikelets. Application of IAA significantly increased the maximum endosperm cell number, grain weight, and the active filling period. In contrast, ABA treatment significantly decreased the concentrations of IAA, GA_1+4_, and Z+ZR and shortened the active filling period, whereas it increased the ABA concentrations, mean filling rate, maximum endosperm cell number, and grain weight of the inferior spikelets (Table 2 and S1 Table).

**Table 2:** Effect of exogenous application IAA and ABA on maximum endosperm cell number, mean grain filling rate, active grain filling period, and maximum grain weight in inferior spikelets.

Cultivar	Treatment	Maximum endosperm cell number (×10^−3^ cells per endosperm)	Mean grain filling rate (mg kernel^-1^ d^-1^)	Active grain filling period (d)	Maximum grain weight (mg kernel^-1^)
AZU-WT	CK	404 ± 4.39c	0.8 ± 0.02b	19.4 ± 0.49b	15.5 ± 0.33c
	20×10^−5^ M IAA	411 ± 4.73b	0.72 ± 0.02c	23.5 ± 0.55a	16.9 ± 0.12b
	25×10^−6^ MABA	419 ± 6.59a	1.16 ± 0.03a	15.1 ± 0.38c	17.5 ± 0.22a
ZF802-WT	CK	223 ± 2.06c	0.70 ± 0.03b	24.3 ± 0.57b	17.0 ± 0.33c
	20×10^−5^ M IAA	227 ± 2.09b	0.62 ± 0.02c	28.4 ± 0.50a	17.6 ± 0.23b
	25×10^−6^ MABA	234 ± 2.46a	0.85 ± 0.03a	21.5 ± 0.40c	18.3 ± 0.25a

Two wild types, AZU-WT and ZF802-WT, were grown in field. Endosperm cell number was measured at 4 d intervals from anthesis to 28 DPA, and grain weight was measured at 4 d intervals from anthesis to maturity. Data are means±SE of six independent measurements and different letters indicate statistical significance at the *P* = 0.05 level within the same column and within the same cultivar.

The application of Put, Spd, and Spm significantly increased the levels of free Put, Spd, and Spm in the inferior spikelets. Application of MGBG to the panicles resulted in a significant increase in the free Put concentration, and significant decrease in the concentrations of free Spd and Spm in all types of spikelets (S2 Table). Chemical treatment did not significantly affect the level of free PAs or the filling characteristics of superior spikelets (Table 3 and S2 Table). When the grains were treated with Spd or Spm, the maximum cell number, the mean filling rate, and the grain weight were significantly increased in inferior spikelets, while treatment with Put had the opposite effects on these parameters (Table 3).

**Table 3:** Effect of exogenous application Put, Spd, Spm and MGBG on maximum endosperm cell number, mean grain filling rate, active grain filling period, and maximum grain weight in both superior and inferior spikelets.

Spikelet type	Cultivar	Treatment	Maximum endosperm cell number (×10^−3^ cells per endosperm)	Mean grain-filling rate (mg kernel^-1^ d^-1^)	Active grain-filling period (d)	Maximum grain weight (mg kernel^-1^)
Superior	AZU-WT	CK	628 ± 8.32a	1.51 ± 0.04a	15.6 ± 0.57b	23.6 ± 0.60 a
		2mM Put	628 ± 8.32 a	1.48 ± 0.03a	15.8 ± 0.53b	23.4 ± 0.57 a
		1mM Spd	630 ± 4.21a	1.52 ± 0.05 a	15.5 ± 0.49b	23.6 ± 0.67a
		1mM Spm	633 ± 8.38 a	1.52 ± 0.04a	15.6 ± 0.60b	23.7 ± 0.71a
		5mM MGBG	609 ± 8.06b	1.23 ± 0.03b	17.9 ± 0.59a	22.0 ± 0.62b
	ZF802-WT	CK	250 ± 5.00a	1.68 ± 0.05 a	11.1 ± 0.31b	18.6 ± 0.74 a
		2mM Put	249 ± 3.16a	1.66 ± 0.04a	11.0 ± 0.37b	18.3 ± 0.69a
		1mM Spd	252 ± 3.50a	1.69 ± 0.05 a	11.1 ± 0.28b	18.7 ± 0.70 a
		1mM Spm	251 ± 3.67a	1.69 ± 0.05a	11.1 ± 0.33b	18.8 ± 0.57a
		5mM MGBG	241 ± 3.78b	1.45 ± 0.02b	12.1 ± 0.34a	17.5 ± 0.50 b
Inferior	AZU-WT	CK	417 ± 7.68b	0.80 ± 0.04b	19.3 ± 0.46b	15.5 ± 0.40b
		2mM Put	392 ± 8.12c	0.64 ± 0.03 c	22.3 ± 0.80a	14.3 ± 0.37c
		1mM Spd	433 ± 9.50a	0.89 ± 0.03a	19.0 ± 0.72c	16.9 ± 0.54 a
		1mM Spm	430 ± 8.91a	0.90 ± 0.04a	19.0 ± 0.54c	17.1 ± 0.41a
		5mM MGBG	351 ± 7.27d	0.57 ± 0.02d	23.2 ± 0.88a	13.2 ± 0.37d
	ZF802-WT	CK	229 ± 3.88b	0.71 ± 0.04b	23.9 ± 0.68c	17.0 ± 0.48b
		2mM Put	211 ± 3.65c	0.61± 0.02c	26.7± 0.76b	16.3 ± 0.46c
		1mM Spd	239 ± 4.95a	0.85 ± 0.03a	21.1 ± 0.70d	17.9 ± 0.51a
		1mM Spm	240 ± 4.01a	0.86 ± 0.05a	20.9 ± 0.59d	18.0 ± 0.57a
		5mM MGBG	192 ± 3.98d	0.53 ± 0.02d	28.7 ± 0.81a	15.2 ± 0.43d

Two wild types, AZU-WT and ZF802-WT, were grown in field. Endosperm cell number was measured at 4 d intervals from anthesis to 28 DPA, and grain weight was measured at 4 d intervals from anthesis to maturity. Data are means±SE of six independent measurements and different letters indicate statistical significance at the *P* = 0.05 level within the same column and within the same cultivar

## Discussion

Previous studies on the grain filling process in rice mainly focused on the comparison between the spikelets at different locations on a panicle of the same or different varieties. However, it was difficult to clarify its physiological and biochemical mechanisms from such studies due to the differences in genetic background or growth and development of different rice varieties. A more systematic analysis is required to study the relationship between grain filling characteristics and endogenous hormones. This study used large- and small-grain mutants with extreme sink sizes as well as their wild-type varieties (control), which could compare grain filling process between mutants (synchronous grain filling) and wild types (asynchronous grain filling) and study the two spikelet types of the same genotype. Our results showed that the filling rate, cell numbers, and division rate did not significantly differ between the spikelets in both rice mutants. However, significant differences were observed for the abovementioned parameters between the two types of spikelets in the grain of the two wild types, indicating that the superior spikelets filled faster and had a larger grain weight than inferior ones (Figs 1 and 2).

Hormones play important roles in plant growth and development [33]. In our study, we demonstrated that in the wild-type grains, the concentrations of plant hormones (IAA, Z+ZR, and ABA) initially increased, peaked at 9–21and 24–27 DPA for the upper and lower spikelets respectively, then declined gradually until the end of the filling period (Figs 4–6). Furthermore, the superior spikelets with higher hormone concentrations (IAA, Z+ZR, and ABA) exhibited higher endosperm cell division and filling rates in the wild-type grains. In contrast, the inferior spikelets showed the lowest hormone concentrations, and the slowest filling rate and rate of cell division in the wild types. For the two mutants, the concentration of these hormones showed slight differences between superior and inferior spikelets, and grain filling rate and rate of cell division were synchronous between the two types of spikelets. Furthermore, regression analysis revealed significant and positive correlations between the filling rate and cell division rate and the hormone concentrations (Z+ZR, IAA, and ABA; Fig 8). When exogenous IAA and ABA were applied to the panicles at 2–6 DPA, both the maximum endosperm cell number and the grain weight were significantly increased (Table 3). These results suggest that hormone levels mediate endosperm cell division, and consequently, improve the rate of filling and weight of grains. In contrast, reduced hormone levels, which were observed in the inferior spikelets, may decrease the grain weight by inhibiting cell division.

We found two sharp contrasts in the patterns between the GAs (GA_1+4_) and the levels of the studied hormones during the grain filling period (Fig 3). The GAs (GA_1+4_) concentrations in both the types of spikelets were the highest at the early filling stage, which temporally relate to the period of rapid embryo enlargement [34]. Regression analysis showed no significant correlation between the filling and cell division rates with the GA_1+4_ concentrations (Fig 8). Furthermore, chemical treatment with GAs at the early filling stage did not significantly affect the cell number or grain weight [35]. These results suggest that GAs may not regulate cell division of endosperm during filling, although they may play an important role in embryogenesis in rice.

Little is known about the effect of PAs on endosperm cell division and grain filling. It is suggested that both soluble- and insoluble-conjugated PAs remain unchanged throughout the grain filling period; however, the observed changes in free PAs and the activities of enzymes that are involved in PA synthesis in the spikelets have been reported to be closely associated with the cell division and filling rates [36]. Our results demonstrated that changes in the free Put, Spd, and Spm concentrations in grains followed a similar pattern as the cell division and filling rates (Figs 1, 2 and 7). Moreover, the concentrations of these PAs were higher in the superior spikelets than in the inferior ones in all the rice genotypes in this study from 2–6 DPA, whereas the results were reversed during the middle and late filling stages. The maximum concentrations of free Spd and Spm were higher in the superior spikelets than in the inferior ones in all the four rice genotypes (Fig 7e–7l). Free Put, Spd, and Spm concentrations were very significantly and positively correlated with the rates of endosperm cells division and filling (Fig 9). Treatment with Spd or Spm during 2–6 DPA resulted in significant increases in the maximum cell number, mean filling rate, and maximum grain weight in inferior spikelets, whereas the results were reversed when MGBG, an inhibitor of Spd and Spm synthesis, was applied to panicles (Table 3). These results indicate that higher concentrations of PAs (Put, Spd and Spm) enhance endosperm cell division and filling. The reduced concentrations of Put, Spd, and Spm may be at least partly responsible for the lower filling rate and grain weight of inferior spikelets.

It is noteworthy that the maximum free Put concentration was much higher in the inferior spikelets than in superior ones, and the two wild types with lower filling rate in inferior spikelets showed higher free Put concentrations (Figs 2 and 7). Treatment with exogenous Put or MGBG to panicles at 2–6 DPA resulted in a significant increase in the free Put concentration, whereas the maximum endosperm cell number, mean filling rate, and maximum weight of the grain were significantly decreased in inferior spikelets (Table 3 and S2 Table). These results may imply that a higher Put concentration in grains would adversely affect the endosperm cell number, filling rate, and grain weight, and an excessive accumulation of Put in plant organs could inhibit growth and development [37].

Plant hormones can act either synergistically or antagonistically and it is the balance between promoting and inhibiting agents that ultimately determines plant growth and development [38, 39]. It is proposed that increases in Spd and Spm biosynthesis are likely to affect the rates of ethylene synthesis since higher PAs (Spd and Spm) and ethylene share a biosynthetic precursor *S*-adenosyl-L-methionine (SAM) [40–42]. There are reports showing that high levels of ethylene and 1-aminocylopropane-1-carboxylic acid (ACC) are closely associated with fruit abortion, low grain filling rate, low grain plumpness and weight in cereal [43, 44]. Our early works showed that PAs (spd, spm) and ABA can interact / antagonize with ethylene respectively, and mediate spikelet development considerably [44, 45].

In addition, we also observed that the change in ABA contents in the grains followed a similar pattern to endosperm cell division rate and grain filling rate, but GAs (GA_1_ + GA_4_) contents were high at the early grain filling stage, and greater in the inferior than in superior spikelets (Figs 1–3 and 6). The endosperm cell division rate and grain filling rate were significantly and positively correlated with the ABA contents, whereas weakly correlate with GAs (GA_1_ + GA_4_) (Fig 8). Application of ABA to panicles significantly decrease the concentrations of GA1+4, whereas increased the concentrations of ABA and rates of cell division and grain filling and grain weight of inferior spikelets (Table 2 and S1 Table). We speculate that an antagonistic interactions between ABA and GAs may be involved in mediating grain filling. It is possible that the lower ABA and higher GAs content leads to the poor grain filling of inferior spikelets.

We observed that changes in IAA content in the grains were similar to the changing pattern of Z + ZR (Figs 4 and 5). The cell division rate and grain filling rate were significantly correlated with IAA content in the grains during the grain filling period (Fig 8). The results support the hypothesis that auxin may stimulate cell division in combination with cytokinins [46]. High auxin levels in the sink could create an ‘attracting power’, leading to increased cytokinin levels in the grain [47, 48].

## Conclusion

The rates of filling and endosperm cell division did not significantly differ between superior and inferior spikelets for the two mutants. However, these features significantly differed between the two types of spikelets for the two wild types. Superior spikelets filled at a faster rate and showed a larger grain weight than the inferior ones. Changes in concentrations of the IAA, Z+ZR, ABA, and PAs (spermidine and spermine) were consistent with the rates of endosperm cell division and grain filling of both types of spikelets for both wild types and mutants. Exogenous chemical application verified the roles of IAA, ABA, and PAs in grain filling. Poor filling in the inferior spikelets in rice is mainly due to the lower Z+ZR, IAA, ABA, and PA concentrations in the grains, leading to a smaller division rate of endosperm cells, fewer endosperm cells, and slower grain filling rate, and accordingly, smaller grain weight.

## Supporting Information

S1 Table Effect of exogenous application IAA and ABA on Z+ZR, GA_1+4_, IAA, ABA concentrations (μg g^-1^ FW) in inferior spikelets. (DOCX)

S2 Table Effect of exogenous application Put, Spd, Spm and MGBG on PAs concentrations (nmol g^-1^ FW) in both superior and inferior spikelets. (DOCX)
