# Supplementary material for: Grain Filling Characteristics and Their Relations with Endogenous Hormones in Large- and Small-Grain Mutants of Rice
Source: PLoS One. 2016 Oct 25;11(10):e0165321. doi: 10.1371/journal.pone.0165321 (PMC5079568; doi:10.1371/journal.pone.0165321)
Supplement: S1 Table — (DOCX) [file pone.0165321.s001.docx]

**S1 Table.** Effect of exogenous application IAA and ABA on Z+ZR, GA_1+4_, IAA, ABA concentrations (µg g^-1^ FW) in inferior spikelets.

| Cultivar | Treatment | Z+ZR | GA1+4 | IAA | ABA |
| --- | --- | --- | --- | --- | --- |
| AZU-WT | CK | 6.6 ± 0.4b | 213 ± 9.1b | 66.0 ± 3.2b | 6.5 ± 0.4b |
|  | 20×10^-5^ M IAA | 7.1 ± 0.4a | 234 ± 9.9a | 91.0 ± 4.4a | 5.3 ± 0.3c |
|  | 25×10^-6^ M ABA | 5.4 ± 0.3c | 186 ±7.9c | 51.0 ± 2.4c | 11.6 ± 0.6a |
|  |  |  |  |  |  |
| ZF802-WT | CK | 5.3 ± 0.3b | 189 ± 9.6b | 34.7 ± 1.4b | 2.5 ± 0.2b |
|  | 20×10^-5^ M IAA | 6.8 ± 0.4a | 220 ± 11.1a | 79.2 ± 3.1a | 1.4 ± 0.1c |
|  | 25×10^-6^ M ABA | 4.7 ± 0.3c | 153 ± 7.8c | 26.1 ± 1.0c | 4.1 ± 0.3a |

Two wild types, AZU-WT and ZF802-WT, were grown in field. Each data was from the average of three determinations at 12, 15 and 18 DPA. Data are means±SE of eighteen independent measurements and different letters indicate statistical significance at the *P*=0.05 level within the same column and within the same cultivar.
