# Supplementary material for: Grain Filling Characteristics and Their Relations with Endogenous Hormones in Large- and Small-Grain Mutants of Rice
Source: PLoS One. 2016 Oct 25;11(10):e0165321. doi: 10.1371/journal.pone.0165321 (PMC5079568; doi:10.1371/journal.pone.0165321)
Supplement: S2 Table — (DOCX) [file pone.0165321.s002.docx]

**S2 Table.** Effect of exogenous application Put, Spd, Spm and MGBG on PAs concentrations (nmol g^-1^ FW) in both superior and inferior spikelets.

| Cultivar | Treatment | Superior | | |  | Inferior | | |
| --- | --- | --- | --- | --- | --- | --- | --- | --- |
|  |  | Put | Spd | Spm |  | Put | Spd | Spm |
| AZU-WT | CK | 114 ± 5.07b | 127 ± 10.59a | 145 ± 9.83a |  | 167 ±11.65c | 90 ± 4.11b | 110 ±5.78b |
|  | 2mM Put | 116 ± 5.34b | 133 ± 11.87a | 147 ± 11.71a |  | 223 ± 10.31a | 87 ± 3.97b | 112 ± 5.88b |
|  | 1mM Spd | 116 ± 5.33b | 134 ± 9.08a | 151 ± 11.63a |  | 164 ±11.43c | 123 ± 5.62a | 115 ± 6.03b |
|  | 1 mM Spm | 111 ± 6.19b | 123 ± 9.82a | 151 ± 10.29a |  | 159 ±16.65c | 87 ± 3.97b | 143 ± 7.50a |
|  | 5mM MGBG | 132 ± 6.06a | 102 ± 4.69b | 121 ± 7.12b |  | 207 ± 9.57b | 56 ± 2.56c | 52 ± 2.73c |
|  |  |  |  |  |  |  |  |  |
| ZF802-WT | CK | 142 ± 8.59b | 84 ± 5.39a | 132 ± 13.48a |  | 156 ± 13.56c | 89 ± 4.09b | 120 ± 5.75b |
|  | 2mM Put | 143 ± 8.65b | 87 ± 7.65a | 135 ± 14.41a |  | 213 ± 15.96a | 87 ± 4.41b | 121 ± 5.83b |
|  | 1mM Spd | 149 ± 5.71b | 90 ± 5.34a | 143 ± 16.30a |  | 144 ± 12.42c | 112 ± 5.14a | 124 ± 5.98b |
|  | 1mM Spm | 146 ± 5.59b | 85 ± 4.94a | 139 ± 15.84a |  | 133 ± 11.47c | 87 ± 4.01b | 149 ± 7.18a |
|  | 5mM MGBG | 213 ± 8.16a | 70 ± 4.01b | 105 ± 9.35b |  | 170 ± 12.47b | 54 ± 2.48c | 60 ± 2.89c |

Two wild types, AZU-WT and ZF802-WT, were grown in field. Each data was from the average of three determinations at 12, 15 and 18 DPA. Data are means±SE of eighteen independent measurements and different letters indicate statistical significance at the *P*=0.05 level within the same column and within the same cultivar.
